# Inflammatory markers in the cerebrospinal fluid linked to mortality in tuberculous meningitis

**DOI:** 10.1093/braincomms/fcaf273

**Published:** 2025-07-16

**Authors:** Sofiati Dian, Valerie A C M Koeken, Edwin Ardiansyah, Ahmad R Ganiem, Kirsten van Abeelen, Raúl Aguirre-Gamboa, Feby Purnama, Sofia Imaculata, Jessi Annisa, Lidya Chaidir, Rovina Ruslami, Leo A B Joosten, Mihai G Netea, Bachti Alisjahbana, Reinout van Crevel, Arjan van Laarhoven, Vinod Kumar

**Affiliations:** Research Centre for Care and Control of Infectious Diseases (RC3ID), Faculty of Medicine, Universitas Padjadjaran, 40161 Bandung, Indonesia; Department of Neurology, Faculty of Medicine, Hasan Sadikin General Hospital, Universitas Padjadjaran, 40161 Bandung, West Java Province, Indonesia; Department of Internal Medicine and Radboud Center for Infectious Diseases (RCI), Radboud Institute for Molecular Life Sciences (RIMLS), Radboud University Medical Center, 6525 GA Nijmegen, The Netherlands; Research Centre for Care and Control of Infectious Diseases (RC3ID), Faculty of Medicine, Universitas Padjadjaran, 40161 Bandung, Indonesia; Research Centre for Care and Control of Infectious Diseases (RC3ID), Faculty of Medicine, Universitas Padjadjaran, 40161 Bandung, Indonesia; Department of Neurology, Faculty of Medicine, Hasan Sadikin General Hospital, Universitas Padjadjaran, 40161 Bandung, West Java Province, Indonesia; Department of Internal Medicine and Radboud Center for Infectious Diseases (RCI), Radboud Institute for Molecular Life Sciences (RIMLS), Radboud University Medical Center, 6525 GA Nijmegen, The Netherlands; Department of Genetics, University Medical Center Groningen, University of Groningen, 9700 RB Groningen, The Netherlands; Research Centre for Care and Control of Infectious Diseases (RC3ID), Faculty of Medicine, Universitas Padjadjaran, 40161 Bandung, Indonesia; Research Centre for Care and Control of Infectious Diseases (RC3ID), Faculty of Medicine, Universitas Padjadjaran, 40161 Bandung, Indonesia; Research Centre for Care and Control of Infectious Diseases (RC3ID), Faculty of Medicine, Universitas Padjadjaran, 40161 Bandung, Indonesia; Research Centre for Care and Control of Infectious Diseases (RC3ID), Faculty of Medicine, Universitas Padjadjaran, 40161 Bandung, Indonesia; Research Centre for Care and Control of Infectious Diseases (RC3ID), Faculty of Medicine, Universitas Padjadjaran, 40161 Bandung, Indonesia; Department of Internal Medicine and Radboud Center for Infectious Diseases (RCI), Radboud Institute for Molecular Life Sciences (RIMLS), Radboud University Medical Center, 6525 GA Nijmegen, The Netherlands; Department of Internal Medicine and Radboud Center for Infectious Diseases (RCI), Radboud Institute for Molecular Life Sciences (RIMLS), Radboud University Medical Center, 6525 GA Nijmegen, The Netherlands; Department for Genomics and Immunoregulation, Life and Medical Sciences Institute (LIMES), University of Bonn, Bonn 53115, Germany; Research Centre for Care and Control of Infectious Diseases (RC3ID), Faculty of Medicine, Universitas Padjadjaran, 40161 Bandung, Indonesia; Department of Neurology, Faculty of Medicine, Hasan Sadikin General Hospital, Universitas Padjadjaran, 40161 Bandung, West Java Province, Indonesia; Department of Internal Medicine and Radboud Center for Infectious Diseases (RCI), Radboud Institute for Molecular Life Sciences (RIMLS), Radboud University Medical Center, 6525 GA Nijmegen, The Netherlands; Centre for Tropical Medicine and Global Health, Nuffield Department of Medicine, University of Oxford, OX3 7LG Oxford, UK; Department of Internal Medicine and Radboud Center for Infectious Diseases (RCI), Radboud Institute for Molecular Life Sciences (RIMLS), Radboud University Medical Center, 6525 GA Nijmegen, The Netherlands; Department of Internal Medicine and Radboud Center for Infectious Diseases (RCI), Radboud Institute for Molecular Life Sciences (RIMLS), Radboud University Medical Center, 6525 GA Nijmegen, The Netherlands; Department of Genetics, University Medical Center Groningen, University of Groningen, 9700 RB Groningen, The Netherlands

**Keywords:** tuberculous meningitis, MMP-10, cerebrospinal fluid, biomarkers for survival, protein QTL

## Abstract

This study examines the role of host inflammation in the high mortality of tuberculous meningitis (TBM) and identifies potential biomarkers associated with improved survival. We conducted a case-control study involving 131 patients in a discovery cohort, 81 TBM patients in a validation cohort, and 43 non-infected controls from a referral hospital in Indonesia. We measured 94 inflammation-related proteins in cerebrospinal fluid (CSF) and performed genome-wide quantitative trait loci (QTL) mapping. Sixty-seven proteins were found to be differentially expressed between TBM patients and controls, with 64 proteins elevated in patients. Five proteins, including vascular endothelial growth factor (VEGF) and matrix metalloproteinase-10 (MMP-10), were identified as predictors of 180-day mortality in TBM patients. The validation cohort confirmed that MMP-10, but not VEGF, was predictive of mortality. Genome-wide QTL mapping identified two genome-wide significant and four suggestive genetic loci associated with CSF MMP-10, which also predicted survival in an additional cohort of 218 patients. High CSF concentrations of MMP-10, along with specific genetic loci, may be associated with survival in TBM patients, suggesting a potential role for MMP-10 in disease pathogenesis and warranting further investigation into its utility in host-directed therapies.

## Teaser

This study explores the role of inflammation in tuberculous meningitis mortality and identifies potential biomarkers for survival. A case-control study was conducted with 131 tuberculous meningitis patients and 43 controls, measuring inflammation-related proteins in cerebrospinal fluid. Matrix metalloproteinase-10 was identified as a predictor of 180-day mortality.

## Graphical Abstract

**Graphical Abstract:**
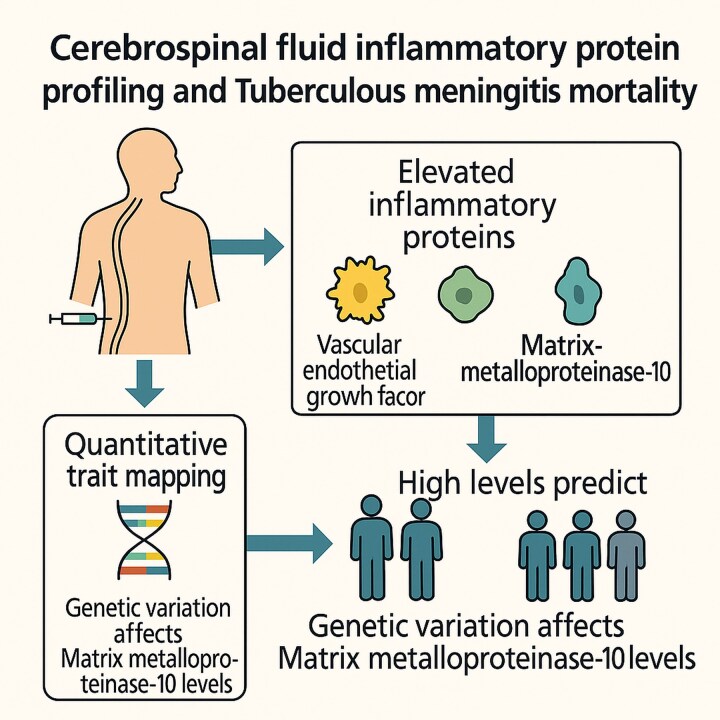

## Introduction

Tuberculous meningitis (TBM) is the most devastating form of tuberculosis, resulting in death or severe disability in approximately half of all patients.^1^ Understanding the factors that affect mortality is crucial for developing new therapeutic strategies. An ineffective host immune system contributes to the poor outcome of TBM, either through inadequate killing of *Mycobacterium tuberculosis*, or through an inappropriate inflammatory response leading to tissue damage (immunopathology).^2^ Corticosteroids, aimed at limiting damaging inflammation, have been shown to reduce patient mortality.^3^ However, disability is not prevented with corticosteroids, and some patients still develop severe immunopathology despite corticosteroid treatment, underlining the need for additional host-directed therapies. Profiling inflammatory proteins in the cerebrospinal fluid (CSF) could help unravel the mechanisms of immunopathology and identify novel therapeutic targets. Yet, studies examining individual inflammatory mediators in relation to clinical outcomes have not shown consistent results. For example, in a prospective study, high IFN-γ in CSF predicted a favourable outcome in HIV-negative patients, but not with HIV,^4^ while another study reported the opposite.^5^

Therefore, the aim of this study was to obtain a broader characterization of the inflammatory response in CSF in TBM using an unbiased proteomics approach, and to evaluate the association between these inflammatory markers and mortality. To this purpose, we measured a large panel of inflammatory proteins in CSF and linked their concentrations to survival in a prospective cohort of TBM patients. Subsequently, we validated our findings in a second cohort of TBM patients. Finally, we investigated whether single nucleotide polymorphisms (SNPs) associated with CSF proteins that predicted survival (i.e. protein quantitative trait loci (QTLs)) were also predicted survival in an independent set of TBM patients.

## Materials and methods

### Study design and participants

Study participants with suspected meningitis were included in a prospective cohort from the Hasan Sadikin Hospital (Bandung, Indonesia) from 31 October 2006 to 16 June 2016.^6^ Patients were diagnosed as definite TBM cases with a CSF culture or PCR positive for *M. tuberculosis*, and as probable TBM cases when they presented with clinically suspected disease, a CSF leukocyte count ≥5 per μL and a CSF:blood glucose-ratio lower than 0.5.^6^ To ensure consistent clinical and laboratory handling procedures, controls were selected from patients who presented with symptoms mimicking meningitis but were ultimately diagnosed with non-infectious conditions, such as metabolic abnormalities. These controls had a negative *M. tuberculosis* culture and normal routine CSF characteristics: CSF leukocyte count <5 per μL and a CSF:blood glucose-ratio higher than 0.5. HIV co-infection, which significantly impacts immune response and mortality of TBM, was an exclusion criterion for the study.

All participants underwent a comprehensive clinical evaluation, including a detailed medical history, general physical examination, and neurological examination. Neurological findings, such as signs of meningeal irritation (e.g. neck stiffness, Kernig’s sign and Brudzinski’s sign), cranial nerve deficits, focal neurological deficits, and altered mental status, were systematically recorded. CSF samples were collected at the time of diagnosis and analysed for routine parameters, including white cell count (total and differential counts for mononuclear and polymorphonuclear cells), protein concentration, and glucose levels. Gram staining and cryptococcal antigen testing were performed to exclude bacterial and cryptococcal meningitis, respectively. Acid-fast bacilli staining, Gene-Xpert, and culture were conducted to confirm *M. tuberculosis* infection. From the prospective cohort, 131 TBM patients and 43 controls were selected as the discovery cohort for proteomic analyses, and an additional 81 TBM patients were included for validation. Genotype data from 209 patients were used for QTL mapping, and genetic validation was performed in another 218 TBM patients. Patients were included under the project ‘Optimization of Diagnosis of Meningitis’, approved by the Ethical Committee of Hasan Sadikin Hospital/Faculty of Medicine of Universitas Padjadjaran, Bandung, Indonesia (449/UN6.C1.3.3/KEPK/PN/2015).

### Protein analysis

CSF samples were centrifuged for 15 min at 1600 × g and the supernatants were stored at −80°C.^7^ The commercially available ProSeek Multiplex Inflammation I panel (Olink Proteomics, Uppsala, Sweden) was used to measure a panel of 92 inflammation-related proteins in CSF. The procedure of the multiplex proximity extension assay was performed as previously described.^8^ Briefly, proteins are recognized by pairs of antibodies coupled to cDNA strands. These cDNA strands bind when they are in close proximity, after which they extend by a polymerase reaction. After detection and normalization, this results in normalized protein expression values, measured on a log_2_-scale. Proteins were excluded from the analysis when the target protein was detected in <75% of the samples. To validate our findings on vascular endothelial growth factor (VEGF), VEGF was measured in CSF samples of a larger validation cohort using the Simple Plex cartridges run on the Ella platform (ProteinSimple, San Jose, California, USA) following the manufacturer’s instructions. Additionally, levels of neuron-specific enolase (NSE), calprotectin (S100A8/A9), and IL-1Ra were measured in these CSF samples using enzyme-linked immunosorbent assays (ELISAs) and were performed according to the manufacturer’s protocol (R&D Systems, Minneapolis, Minnesota, USA). Ella and ELISA data were log-transformed before analysis. Serum and CSF albumin were measured in a subgroup of patients as previously described.^9^

### Protein clustering and differential abundance analyses

Data analyses of this study included protein, survival, and QTL analysis. Protein values under the detection threshold were replaced with the lower limit of detection values. All computational analyses were performed in R 4.3.3. Protein levels were compared between TBM patients and controls using two-sample Wilcoxon tests. Proteins levels were then correlated using Spearman’s Rank-Order correlation and clustered using average hierarchical clustering using a maximum distance between proteins of 0.3, visualized using the R package ‘corrplot’. Each cluster was represented by the protein with the lowest distance to the other proteins in that cluster. Due to high correlation between markers and to avoid the burden of multiple testing corrections, only the representatives were included in further analysis, which enabled us to analyse groups of proteins rather than all markers individually.

### Statistical analysis

The protein cluster representatives were correlated to relevant measures of inflammation or brain damage using Spearman correlation and analysed for survival using Cox regression with R packages ‘survival’ and ‘survminer’. A false discovery rates (FDR) based on Benjamini–Hochberg procedure of <0.05 was considered significant. The proteins that significantly predicted survival in the discovery cohort were then tested in the validation cohort.

### Genome-wide QTL mapping

Genotyping was performed as previously described.^9^ For imputation, we utilized an Indonesian-specific reference panel, which was created by incorporating whole genome sequence data from 217 Indonesian samples into the publicly available 1000 Genomes Project Phase 3 (1KGP3) East Asian reference panel.^10^ Briefly, 4 125 096 SNPs with an imputation score (*R*^2^) >0.8 and minor allele frequency ≥0.05 were included in the analysis. To identify QTLs, log_2_-normalized protein expression values for CSF proteins were mapped to the genotype data using a linear regression model with the R package ‘MatrixEQTL’. Visualization of the Manhattan plot was performed using ‘qqman’. Sex and age were included in the analysis as covariates. The threshold for genome-wide significance was set at *P* < 5 × 10^−8^, and *P* < 1 × 10^−5^ was considered as a suggestive association. We calculated a genetic risk score based on the QTLs with a *P* < 1 × 10^−5^ using the linear component of the Cox model, as in genetic risk score = β_1_×_1_+β_2_×_2_+…+β_p_x_p_, where x_1_ is the genotype data in dosages and β_1_ is the coefficient resulting from fitting the Cox proportional model.^9^ The genetic patient cohort was stratified into high and low risk groups according to the median of the genetic risk score, and related to mortality.

## Results

### Inflammation-related proteins in the CSF of tuberculous meningitis patients

**Figure 1:**
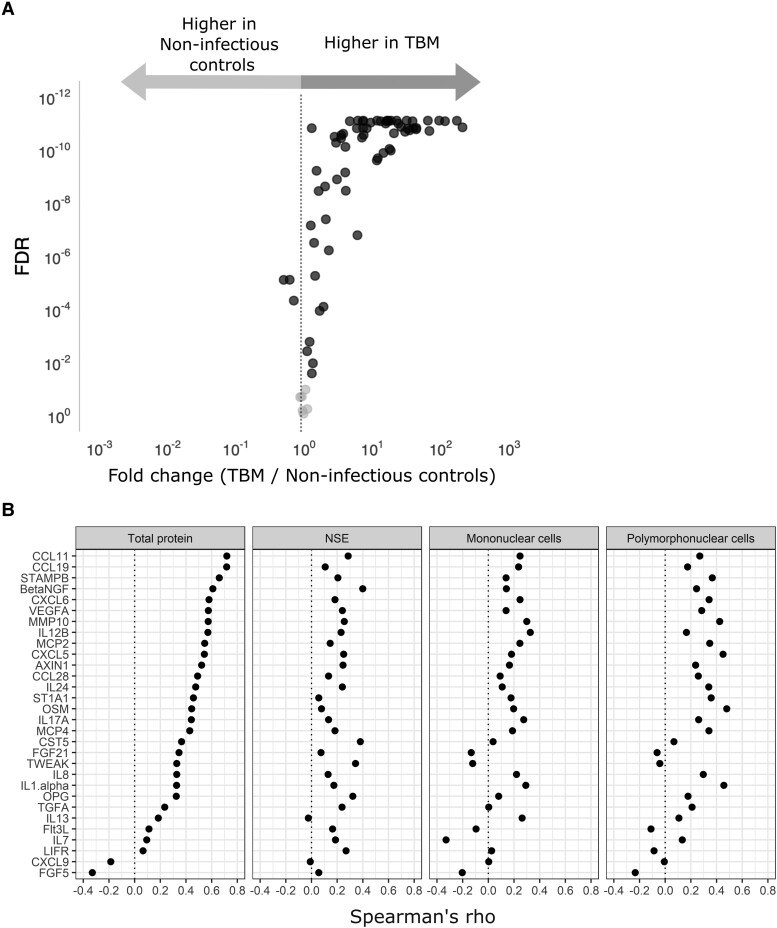
**Protein levels show large differences between patients and controls in CSF.** (**A**) Volcano plot for individual proteins in CSF with the fold change between TBM and non-infectious controls on the *x*-axis, and the FDR on the *y*-axis. Proteins significantly different between TBM and controls (FDR < 0.05) are coloured in black (*N* = 67), while the statistically insignificant proteins are coloured in grey (*N* = 3). Each point represents one protein. (**B**) Correlation plots for representatives of CSF protein clusters with CSF total protein (as a proxy for barrier disruption) for 131 TBM patients, NSE (as proxy for brain damage), and CSF mononuclear and polymorphonuclear cell counts. Each data point on the *x*-axis represents the Spearman’s rho value for a single protein.

For the proteomic analysis, 131 TBM patients and 43 controls were included. Of those 131 TBM patients, 97 (74%) had definite TBM, as confirmed by CSF culture or PCR. The patient characteristics of the cohort are summarized in Supplementary Tables 1 and 2. In total, 94 inflammatory proteins were measured in CSF. The quality of the measurement was high and 99% of the samples passed quality control. Overall, 70 of the 94 (74%) proteins were detected in at least 75% of the TBM patients’ CSF samples and were included in downstream analysis. TBM patients had a distinct CSF protein profile compared to controls. Of the 70 proteins, 67 (96%) differed significantly between the controls and the TBM group, with 64 proteins showing higher and 3 showing lower concentrations in TBM patients compared to controls (Fig. 1A and Supplementary Table 3).

Subsequently, we analysed global correlation patterns among CSF proteins in TBM patients. Using Spearman correlations and average hierarchical clustering, we identified 24 clusters of proteins, each represented by the protein with the lowest relative distance to others in the cluster (Supplementary Fig. 1). Most protein pairs showed positive correlations, although proteins such as C-X-C motif ligand (CXCL) 9, CXCL10 and fibroblast growth factor 5 showed mainly inverse correlations with the other proteins. None of the CSF protein concentrations correlated well (Spearman’s rho >0.5) with CSF mononuclear cell count or polymorphonuclear cell counts, indicating that CSF leukocytes are probably not the main source of these proteins. Four (17%) protein cluster representatives showed strong correlations (Spearman’s rho >0.5) with NSE, a well-established marker for neuronal damage.^11^ Correlations were especially high for CSF total protein (10 representatives with rho >0.5) (Fig. 1B and Supplementary Table 3), which can serve as a proxy for blood–CSF barrier disruption (correlation with CSF-serum albumin gradient; Pearson *r* = 0.95, *P* < 0.0001).^12^

### CSF matrix metalloproteinase-10 concentrations predict patient survival

Among 131 TBM patients followed until 6 months (94% complete follow-up), the mortality rate was 44%. Five protein cluster representatives significantly predicted survival in a Cox regression model after correction for multiple testing (FDR < 0.05). All five had hazard ratios above 1, indicating that higher CSF protein concentrations were associated with increased mortality. These five CSF proteins were also measured in the validation cohort of 81 TBM patients to assess whether they similarly predicted survival (Table 1).

**Table 1:** Cox regression for 180-day mortality for the protein cluster representatives

	Discovery cohort				Validation cohort		
Representative	Hazard ratio	+ 95% CI	*P*-value	FDR	Hazard ratio	+ 95% CI	*P*-value
TWEAK	1.93	(1.37–2.72)	1.55 × 10^−4^	1.92 × 10^−3^	1.02	(0.58–1.78)	0.948
VEGF	1.57	(1.24–1.99)	1.62 × 10^−4^	1.92 × 10^−3^	1.23	(0.86–1.75)	0.252
OPG	1.49	(1.18–1.88)	6.85 × 10^−4^	5.52 × 10^−3^	1.15	(0.86–1.54)	0.354
IL-7	1.60	(1.16–2.21)	3.92 × 10^−3^	2.35 × 10^−2^	1.73	(0.94–3.19)	0.079
MMP-10	1.31	(1.08–1.60)	6.11 × 10^−3^	2.93 × 10^−2^	1.49	(1.09–2.04)	0.012

The hazard ratio is calculated per 2-fold increase. Only the statistically significant protein representatives are included in this table. Discovery cohort, *N* = 130 with 57 events; Validation cohort, *N* = 81 with 26 events. CI, confidence interval; FDR, false discovery rate.

**Figure 2:**
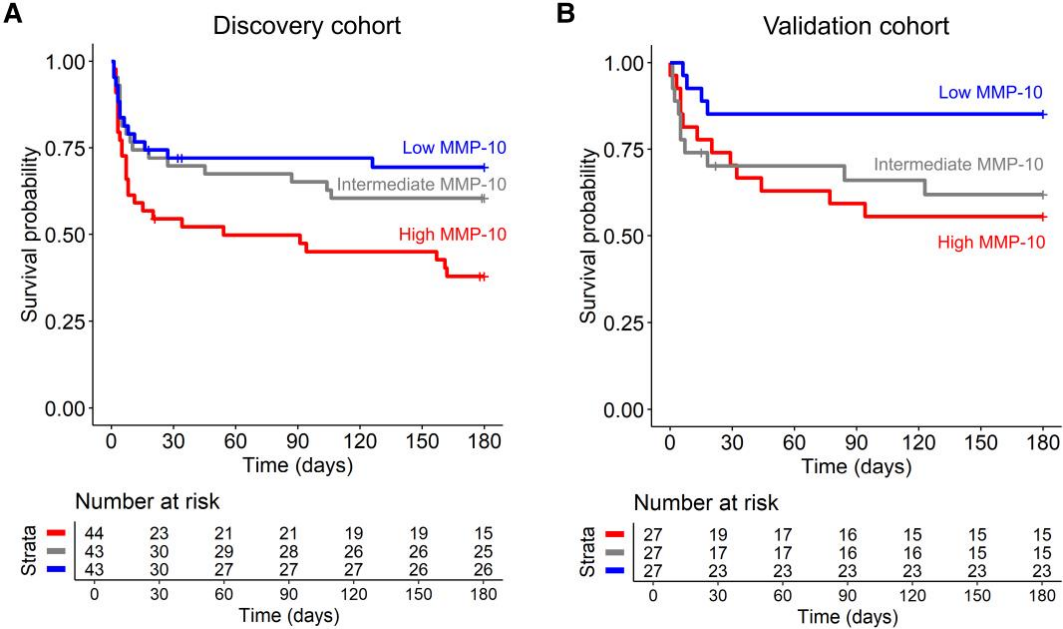
**CSF MMP-10 concentrations predict survival in tuberculous meningitis.** Kaplan–Meier graph with survival table for patients of the protein discovery (*N* = 130, **A**) and the protein validation cohort (*N* = 81, **B**) based on CSF MMP-10 concentrations divided in tertiles.

Of the five markers, matrix metalloproteinase-10 (MMP-10), was significantly associated with mortality in the validation cohort (Table 1 and Fig. 2A and B). A 2-fold increase in CSF MMP-10 levels corresponded to a hazard ratio of 1.31 for 180-day mortality (95% CI 1.08–1.6, *P* = 6.1 × 10^−3^, Fig. 2A). CSF MMP-10 levels also correlated with CSF total protein (Spearman’s rho = 0.57, *P* = 1.2 × 10^−12^), CSF NSE (Spearman’s rho = 0.48, *P* = 4.0 × 10^−7^), CSF polymorphonuclear cell counts (Spearman’s rho = 0.42, *P* = 4.9 × 10^−7^) and CSF mononuclear cell counts (Spearman’s rho = 0.30, *P* = 5.6 × 10^−4^). One of the markers identified in the discovery cohort, VEGF, could not be validated. Given VEGF’s plausible biological role in TBM, we explored whether the lack of validation was due to limited sample size. Therefore, we measured VEGF in a total of 199 TBM patients that were not part of the discovery cohort. However, VEGF did not predict survival in these patients (hazard ratio = 1.03, *P* = 0.51).

### Genetic variation associated with CSF MMP-10 levels

**Figure 3:**
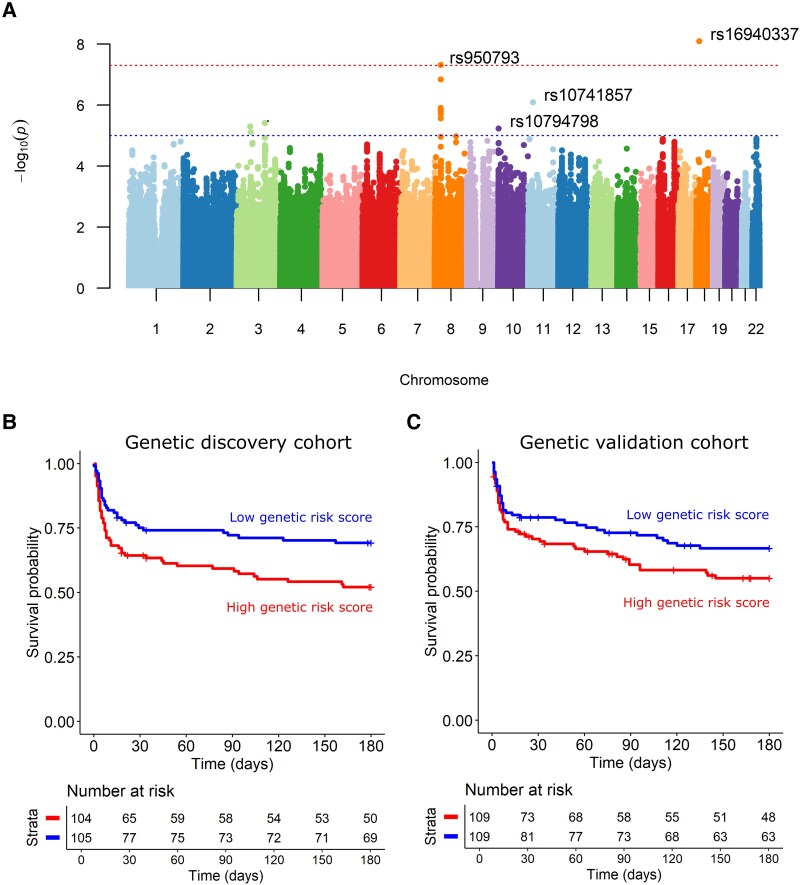
**Genetic variants predict MMP-10 concentrations in CSF from tuberculous meningitis patients.** (**A**) Manhattan plot showing the genome-wide QTL mapping results for CSF MMP-10 concentrations in TBM patients of the genetic discovery cohort (*N* = 209; three patients were excluded from the analysis because of missing genetic data); horizontal lines correspond to *P* < 5 × 10^−8^ and *P* < 1 × 10^−5^. Each data point indicates the −log_10_  *P*-value of a single SNP in relation to MMP-10 protein levels. (**B** and **C**) Survival according to the genetic risk score for the genetic discovery cohort (**B**, *N* = 209), and the genetic validation cohort (**C**, *N* = 218).

To investigate the genetic regulation of MMP-10 levels in CSF, we performed genome-wide association analysis by correlating SNP genotypes with CSF MMP-10 concentrations to identify quantitative trait loci (pQTLs, Fig. 3A). Two loci were associated with MMP-10 levels at genome-wide significant threshold (*P* < 5 × 10^−8^), and four additional loci showed suggestive association (*P* < 1 × 10^−5^; Supplementary Table 4). Given the strong inter-correlation among CSF proteins (Supplementary Fig. 1), we examined whether the identified MMP-10 pQTLs were also associated with other protein cluster representatives that significantly predicted mortality in the discovery cohort (Supplementary Table 5). A few of these loci were shared, particularly with those regulating interleukin-7 (IL-7) and tumour necrosis factor-like weak inducer of apoptosis (TWEAK) (Supplementary Fig. 2).

To understand the possible biological relevance of these QTLs, we explored if these SNPs were known expression QTLs in blood using publicly available data source (HaploReg v4.1).^13^ None of the identified loci were known expression QTLs, suggesting they may exert tissue-specific regulatory effects, possibly in the central nervous system.

### MMP10 QTLs predict survival of TBM patients

We next examined whether the identified QTLs for CSF MMP-10 could together predict patient survival. A genetic risk score was calculated for each patient based on the genotypes at the six loci associated with CSF MMP-10 levels. Patients were then stratified into low-risk and high-risk groups based on the median genetic risk score for the cohort. In the 209 TBM patients included for QTL mapping, patients carrying the ‘favourable’ genotypes, which correlates to low levels of CSF MMP-10, exhibited significantly lower mortality. In contrast, patients with an ‘unfavourable’ genetic make-up, which links to high CSF MMP-10 concentrations, showed higher mortality (*P* = 0.012, Fig. 3B). We attempted to validate this finding using a second group of 218 TBM patients (55% culture-confirmed) with 82 (38%) recorded deaths (Supplementary Table 1). Consistent with the discovery cohort, an ‘unfavourable’ genetic risk score, indicating genotypes with elevated CSF MMP-10 levels, showed a trend of association with higher mortality (*P* = 0.092) (Fig. 3C). This suggests that a patient’s genetic make-up may affect CSF MMP-10 levels, which may in turn contribute to clinical outcome in TBM.

## Discussion

By profiling a large panel of inflammatory proteins in the CSF of HIV-negative TBM patients, we identified five proteins including MMP-10 that were independently associated with mortality. Notably, MMP-10’s predictive value was confirmed in an independent patient cohort, highlighting its potential as a biomarker for TBM prognosis.

MMP-10 (stromelysin-2) is a proteolytic enzyme that degrades multiple components of the extracellular matrix.^14^ It is elevated in induced sputum and bronchoalveolar lavage fluids of pulmonary tuberculosis patients compared to controls,^15^ and has also been implicated in host immune responses to other pathogens.^16^ Furthermore, in HIV co-infected TBM patients, CSF MMP-10 levels increased before the onset of immune reconstitution inflammatory syndrome following antiretroviral therapy,^17^ suggesting a role in TBM-related immunopathology. As such, MMP-10 has been hypothesized to play a role in immunopathology in the context of tuberculosis but is less studied compared to other MMP’s.^18^ MMP-10 can activate MMP-1, yet MMP-1 was not predictive of mortality in our study, indicating the possible contribution of MMP-10 to mortality through pathways independent of MMP-1 activation. However, since the antibody-based assay used in our study cannot distinguish between the pro-enzyme or the active form of MMP-1, we cannot draw definite conclusions regarding the biological activity of MMP-1.

The blood, brain and CSF are different compartments of the central nervous system, separated by the blood–brain and blood–CSF barriers. The blood–brain barrier is a highly selective barrier essential for maintaining cerebral homeostasis. Disruption of these barriers is a hallmark of neuroinflammation during CNS infections, and *in vitro* studies have implicated MMPs in blood–brain barrier disruption.^19^ Numerous MMPs have been shown to be elevated in TBM^18^ and we observed that CSF MMP-10 concentrations correlated with CSF total protein, a surrogate for blood–CSF barrier dysfunction, which correlates to CSF/serum albumin quotient.^12^ This supports the hypothesis that MMP-10 may contribute to TBM immunopathology by inducing barrier disruption. Of note, MMP-10 also correlated to NSE, which may reflect neuronal destruction. As dexamethasone decreases the secretion of MMP-1, MMP-3 and MMP-9,^20,21^ it should be investigated whether the beneficial effect of corticosteroid treatment in TBM could partially be explained by reducing the expression of MMPs. Then, specific MMP inhibitors could be a promising approach to further reduce mortality.

MMP-10 was strongly associated with TBM mortality and correlated with elevated CSF protein levels, suggesting a broader role in immunopathology. Its correlation with multiple inflammatory proteins and the high inter-protein correlation observed in CSF indicate that MMP-10 may be part of a coordinated inflammatory response rather than acting alone. Shared genetic regulation across several mortality-associated proteins supports the presence of pleiotropic effects, where single variants influence multiple proteins. These trans-regulatory mechanisms may reflect upstream control by immune-related pathways, potentially amplifying inflammation and contributing to disease severity. While these findings highlight the genetic architecture underlying CSF immune responses in TBM, replication in larger, independent cohorts is needed, particularly given the limited sample size and lack of brain-specific regulatory data. Understanding these shared genetic networks may help identify central targets for therapeutic intervention.

Our study has several limitations. We did not include plasma measurements or calculate the albumin quotient to directly estimate blood–brain barrier leakage. Nor did we perform longitudinal sampling, which may have provided better predictive insights. However, previous metabolomic study has shown the CSF metabolome to be more informative than plasma in TBM patients,^9^ leading us to prioritize studying the immune response in the CSF compartment. Also, we could not examine brain tissue to establish the cellular source of detrimental MMP-10 production, because brain autopsy is not part of Indonesian medical practice. In addition, studying members of the matrix metalloproteinase family other than MMP-1 and MMP-10 could provide additional insights in the proteolytic pathways involved.^18^ Although we validated our proteomic findings in a second group of patients, validation of genetic findings was not statistically significant. This needs further study in larger cohorts and as well as in patients from a different geographical setting or genetic background. Lastly, it may be of interest to replicate our findings in other CNS infections.

In conclusion, elevated CSF MMP-10 concentrations are associated with increased mortality in TBM, possibly because of its effect on blood–brain barrier integrity, leading to immunopathology. Our data suggest that CSF protein concentrations in TBM are under strong genetic influence, likely contributing to the variable outcome of TBM. Future studies should examine the possible benefit of targeting MMPs in TBM. Combined with timely diagnosis, appropriate supportive therapy,^22^ and intensified antibiotic treatment^23,24^ these and other strategies to limit immunopathology may help improve outcome of this most deadly manifestation of tuberculosis.

## Supplementary Material

fcaf273_Supplementary_Data

## Data Availability

Proteomics data were generated at OLINK facility. Derived data supporting the findings of this study are available from the corresponding author on request. The genetic data are not publicly available due to privacy reasons of research participants.
