# Supplementary material for: Inflammatory markers in the cerebrospinal fluid linked to mortality in tuberculous meningitis
Source: Brain Commun. 2025 Jul 16;7(4):fcaf273. doi: 10.1093/braincomms/fcaf273 (PMC12284393; doi:10.1093/braincomms/fcaf273)
Supplement: fcaf273_Supplementary_Data [file fcaf273_supplementary_data.pdf]

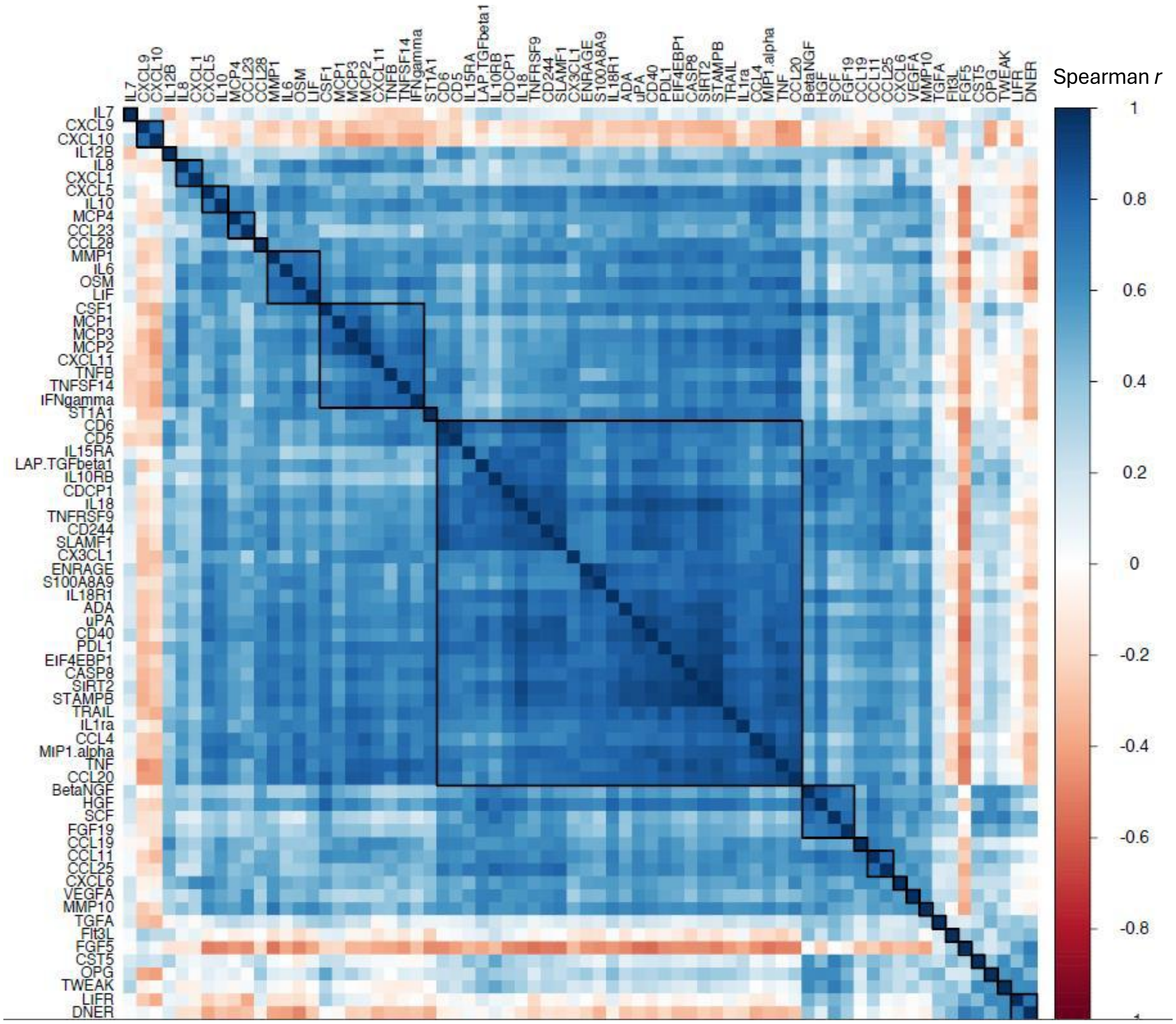

Supplementary Figure 1. High overall correlation between inflammatory proteins.

Correlation matrix of 131 TBM patients based on Spearman correlations, in which the proteins are ordered by average hierarchical clustering. The squares indicate 24 clusters, with a maximum distance between proteins in the same cluster is 0.3.

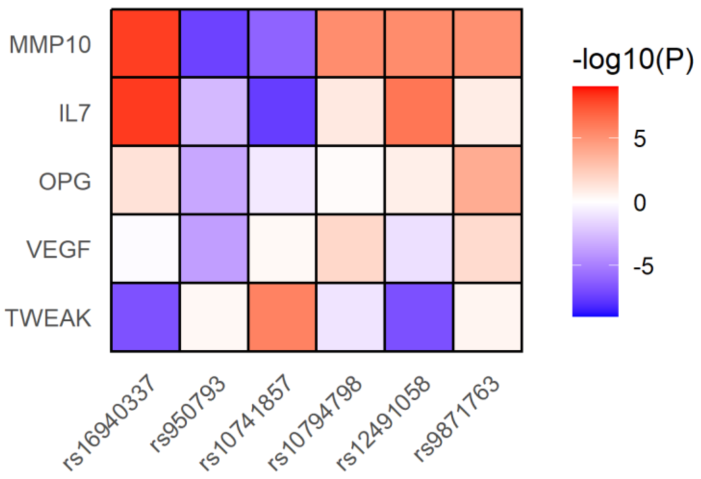

Supplementary Figure 2. MMP-10 QTLs associated with five protein cluster representatives predictive of survival

Heatmap displaying –log10(p-value) of MMP-10 QTLs across five protein cluster representatives in 209 TBM patients. Red indicates positive correlation between proteins and the effect allele, while blue indicates negative correlation.

Supplementary Table 1. Patient characteristics

non	Protein discovery cohort		Protein validation cohort	Genetic validation cohort
	TBM (N = 131)	Non-infectious controls (N = 43)	TBM (N = 81)	TBM (N = 218)
**Clinical parameters**				
Sex, male	82 (63%)	23 (53%)	43 (53%)	116 (53%)
Age, years	29 [22, 38]	35 [22, 42]	31 [23, 38]	27 [21, 36]
Tuberculous meningitis grade				
Grade I	6/130 (5%)	NA	7/73 (10%)	23/199 (12%)
Grade II	102/130 (78%)	NA	56/73 (77%)	154/199 (77%)
Grade III	22/130 (17%)	NA	10/73 (14%)	22/199 (11%)
Temperature, °C	37.7 [37.0, 38.2]	37.1 [36.7, 37.8]	37.5 [36.8, 38.0]	37.6 [36.8, 38.0]
Glasgow Coma Scale	13 [11, 14]	15 [14, 15]	14 [13, 15]	14 [12, 15]
Seizures present	7/126 (6%)	6 (14%)	5/69 (7%)	13/189 (7%)
Motor abnormalities present	73/129 (57%)	24 (56%)	44/69 (64%)	92/185 (50%)
Cranial nerve palsy present	90 (69%)	23/42 (55%)	56/78 (72%)	115/193 (60%)
Chest X-ray abnormal	98/129 (76%)	31 (72%)	58/77 (75%)	138/195 (71%)
**Cerebrospinal fluid parameters**				
Leukocytes, cells/μL	205 [70, 368]	2 [1, 2]	142 [37, 290]	138 [50, 342]
Mononuclear cells, cells/μL	106 [35, 188]	1 [0, 1]	81 [32, 171]	72 [29, 177]
Polymorphonuclear cells, cells/μL	65 [19, 173]	0 [0, 1]	20 [4, 69]	29 [8, 98]
Protein, mg/dL	182 [122, 357]	27 [20, 38]	175 [98, 321]	200 [110, 432]
Neuron-specific enolase, ng/mL	24.7 [15.9, 39.9]	15.5 [10.2, 21.1]	NA	NA
CSF to blood glucose ratio	0.17 [0.10, 0.24]	0.59 [0.54, 0.69]	0.24, [0.14, 0.38]	0.20 [0.11, 0.33]
M. tuberculosis culture positive	97 (74%)	0 (0%)	29 (36%)	117/214 (55%)
**Blood parameters**				
Hemoglobin, g/dL	12.3 [10.5, 13.7]	11.3 [8.9, 12.8]	12.4 [11.0, 13.6]	12.0 [10.4, 13.4]
Leukocytes, x 10^9/L	10.9 [8.8, 13.7]	8.2 [7.0, 12.3]	10.4 [7.4, 14.0]	11.3 [8.1, 14.2]
Thrombocytes, x 10^9/L	302 [229, 382]	254 [168, 333]	284 [218, 383]	300 [217, 373]
**Outcomes**				
Length of hospitalization, days	19 [7, 24]	15 [7, 19]	19 [12, 22]	16 [7, 22]

Outcome at day 180				
Alive	65/130 (50%)	NA	53 (65%)	111 (51%)
Deceased	57/130 (44%)	NA	26 (32%)	82 (38%)
Lost to follow-up	8/130 (6%)	NA	2 (2%)	25 (11%)

Data are presented as N (%) or median ± interquartile range. Abbreviations: CSF = cerebrospinal fluid; NA = not applicable; TBM = tuberculous meningitis. The protein discovery and protein validation cohort jointly formed the genetic discovery cohort.

Supplementary Table 2. Patient characteristics by TBM status

	Protein discovery cohort		Protein validation cohort	
	**Definite TBM (N = 105)**	**Probable TBM (N = 26)**	**Definite TBM (N = 29)**	**Probable TBM (N = 52)**
**Clinical parameters**				
Sex, male	67 (64%)	15 (57.7%)	19 (65.5%)	24 (46.2%)
Age, years	27.00 [21, 34]	36.50 [30, 45]	29 [21, 35]	32.5 [25.25, 46]
Tuberculous meningitis grade				
Grade I	4 (3.8%)	2 (7.7%)	2 (7.4%)	5 (10.9%)
Grade II	81 (77.9%)	21 (80.8%)	23 (85.2%)	33 (71.7%)
Grade III	19 (18.3%)	3 (11.5%)	2 (7.4%)	8 (17.4%)
Temperature, °C	37.65 [37, 38.2]	37.65 [36.9, 38]	37.80 [37.25, 38.10]	37 [36.7, 37.95]
Glasgow Coma Scale	13 [11, 13]	13 [11, 15]	14.00 [13.00, 14.50]	14 [13, 15]
Seizures present	7 (6.9%)	0 (0%)	1 (4.8%)	4 (8.3%)
Motor abnormalities present	59 (57.3%)	14 (53.8%)	14 (60.9%)	30 (65.2%)
Cranial nerve palsy present	79 (75.2%)	11 (42.3%)	20 (74.1%)	36 (70.6%)
Chest X-ray abnormal	81 (78.6%)	17 (65.4%)	17 (65.4%)	41 (80.4%)
**Cerebrospinal fluid parameters**				
Leukocytes, cells/μL	213 [87, 365]	156 [46, 384]	198 [85, 274]	129.5 [25.75, 292]
Mononuclear cells, cells/μL	105 [38, 180]	121 [19, 201]	108.83 [54.99, 192.72]	71.7 [18.1, 137.9]
Polymorphonuclear cells, cells/μL	71 [25, 175]	26.23 [5, 83]	30.30 [6.40, 73.96]	16.86 [3.9, 58.14]
Protein, mg/dL	198 [128, 355]	150 [82, 357]	189 [132, 308]	166 [88.2, 321.7]
CSF to blood glucose ratio	0.16 [0.09, 0.21]	0.25 [0.19, 0.36]	0.15 [0.1, 0.24]	0.25 [0.19, 0.39]
M. tuberculosis culture positive	97 (92.4%)	0 (0%)	29 (100%)	0 (0%)
**Blood parameters**				
Hemoglobin, g/dL	12 [10.5, 13.7]	12.1 [10.2, 13.7]	11.8 [10.8, 13.15]	12.4 [11.1, 13.7]
Leukocytes, x 10^9/L	11.2 [9.1, 13.8]	9.2 [6.62, 11.97]	10.1 [7.4, 12]	10.5 [7.3, 14.9]
Thrombocytes, x 10^9/L	322 [243, 390]	249 [163, 339]	286 [183.5, 359]	283 [219, 388]
**Outcomes**				
Length of hospitalization, days	20 [7, 24]	17 [5, 22]	20 [15, 22]	16 [11, 21]
Outcome at day 180				
Alive	53 (50.5%)	13 (50%)	18 (62.1%)	35 (67.3%)
Deceased	46 (43.8%)	11 (42.3%)	10 (34.5%)	16 (30.8%)
Lost to follow-up	6 (5.7%)	2 (7.7%)	1 (3.4%)	1 (1.9%)

Supplementary Table 3. Overview of proteins measured in cerebrospinal fluid.

Protein	Detected (%)	Uniprot ID	LLOQ (pg/mL)	ULOQ (pg/mL)	Cluster representative	TBM vs. controls		Corr. CSF PMN		Corr. CSF MN		Corr. CSF protein		Corr. CSF NSE		Cox regression survival		
						FC	FDR	R	p	R	p	R	p	R	p	HR	95% CI	p
ADA	100	P00813	0.48	31250	STAMPB	7.42	2.2×10^{-17}	0.36	2.2×10^{-5}	0.17	5.4×10^{-2}	0.58	4.6×10^{-13}	0.52	1.8×10^{-8}	1.16	(0.97-1.38)	1.1×10^{-1}
ARTN	14	Q5T4W7	0.48	31250	NA	NA	NA	NA	NA	NA	NA	NA	NA	NA	NA	NA	NA	NA
AXIN1	28	O15169	61.04	62500	NA	NA	NA	NA	NA	NA	NA	NA	NA	NA	NA	NA	NA	NA
BDNF	12	P23560	-	-	NA	NA	NA	NA	NA	NA	NA	NA	NA	NA	NA	NA	NA	NA
Beta-NGF	99	P01138	0.48	15625	BetaNGF	1.63	8.6×10^{-14}	0.24	5.2×10^{-3}	0.14	1.1×10^{-1}	0.61	1.7×10^{-14}	0.52	2.0×10^{-8}	1.91	(1.09-3.33)	2.3×10^{-2}
CASP-8	94	Q14790	0.48	31250	STAMPB	17.47	7.2×10^{-19}	0.38	7.6×10^{-6}	0.13	1.5×10^{-1}	0.58	6.3×10^{-13}	0.44	3.3×10^{-6}	1.06	(0.92-1.21)	4.5×10^{-1}
CCL11	95	P51671	3.81	31250	CCL11	2.12	7.6×10^{-13}	0.27	2.0×10^{-3}	0.25	4.7×10^{-3}	0.72	2.2×10^{-16}	0.50	9.9×10^{-8}	1.39	(1.05-1.86)	2.4×10^{-2}
CCL19	99	Q99731	15.26	31250	CCL19	20.78	9.3×10^{-18}	0.17	4.8×10^{-2}	0.23	7.1×10^{-3}	0.72	2.2×10^{-16}	0.36	1.9×10^{-4}	0.95	(0.82-1.09)	4.3×10^{-1}
CCL20	92	P78556	7.63	15625	STAMPB	39.28	3.5×10^{-18}	0.40	2.4×10^{-6}	0.25	4.7×10^{-3}	0.66	2.2×10^{-16}	0.44	4.1×10^{-6}	1.03	(0.94-1.12)	5.2×10^{-1}
CCL23	100	P55773	30.52	31250	MCP-4	34.58	1.6×10^{-18}	0.43	2.5×10^{-7}	0.20	2.1×10^{-2}	0.49	3.8×10^{-9}	0.39	6.5×10^{-5}	1.12	(0.98-1.29)	9.9×10^{-2}
CCL25	98	O15444	3.81	62500	CCL11	2.81	5.1×10^{-16}	0.17	4.8×10^{-2}	0.16	6.8×10^{-2}	0.77	2.2×10^{-16}	0.46	1.1×10^{-6}	1.52	(1.21-1.91)	3.1×10^{-4}
CCL28	98	Q9NRJ3	122.07	1000000	CCL28	1.28	4.5×10^{-17}	0.26	3.0×10^{-3}	0.09	3.0×10^{-1}	0.49	3.5×10^{-9}	0.27	5.6×10^{-3}	1.74	(0.41-7.31)	4.5×10^{-1}
CCL4	100	P13236	1.91	31250	STAMPB	18.55	8.3×10^{-19}	0.48	1.0×10^{-8}	0.20	2.2×10^{-2}	0.56	3.6×10^{-12}	0.37	1.2×10^{-4}	1.03	(0.89-1.19)	6.7×10^{-1}
CD244	95	Q9BZW8	0.06	7812	STAMPB	6.16	7.2×10^{-19}	0.27	1.8×10^{-3}	0.23	1.0×10^{-2}	0.80	2.2×10^{-16}	0.49	2.6×10^{-7}	1.2	(0.97-1.49)	8.7×10^{-2}
CD40	100	P25942	0.01	3906	STAMPB	17.68	7.2×10^{-19}	0.37	1.6×10^{-5}	0.10	2.4×10^{-1}	0.68	2.2×10^{-16}	0.53	8.5×10^{-9}	1.23	(1-1.51)	5.0×10^{-2}
CD5	98	P06127	0.12	3906	STAMPB	21.00	7.2×10^{-19}	0.20	2.2×10^{-2}	0.35	3.5×10^{-5}	0.67	2.2×10^{-16}	0.37	1.5×10^{-4}	0.92	(0.79-1.08)	3.2×10^{-1}
CD6	94	Q8WWJ7	0.24	7812	STAMPB	7.43	7.2×10^{-19}	0.21	1.9×10^{-2}	0.28	1.3×10^{-3}	0.73	2.2×10^{-16}	0.41	2.0×10^{-5}	1.09	(0.9-1.33)	3.8×10^{-1}
CDCP1	100	Q9H5V8	0.12	7812	STAMPB	5.99	3.6×10^{-18}	0.27	1.9×10^{-3}	0.19	3.3×10^{-2}	0.78	2.2×10^{-16}	0.55	2.9×10^{-9}	1.21	(0.98-1.48)	7.0×10^{-2}
CSF-1	100	P09603	0.01	1953	MCP-2	2.69	4.5×10^{-17}	0.33	1.0×10^{-4}	0.16	7.3×10^{-2}	0.62	6.7×10^{-15}	0.44	3.4×10^{-6}	1.05	(0.74-1.49)	8.0×10^{-1}
CST5	100	P28325	1.91	15625	CST5	1.10	2.2×10^{-4}	0.07	4.5×10^{-1}	0.04	6.9×10^{-1}	0.36	2.1×10^{-5}	0.29	2.8×10^{-3}	2.75	(0.74-10.26)	1.3×10^{-1}
CX3CL1	100	P78423	15.26	15625	STAMPB	7.44	6.8×10^{-18}	0.27	1.8×10^{-3}	0.16	7.3×10^{-2}	0.59	9.9×10^{-14}	0.49	2.7×10^{-7}	1.07	(0.92-1.25)	3.8×10^{-1}
CXCL1	100	P09341	7.63	15625	IL-8	28.32	1.0×10^{-18}	0.30	4.2×10^{-4}	0.10	2.5×10^{-1}	0.33	1.3×10^{-4}	0.19	5.4×10^{-2}	0.99	(0.85-1.16)	9.3×10^{-1}
CXCL5	100	P42830	0.95	7812	CXCL5	44.75	1.4×10^{-18}	0.45	7.8×10^{-8}	0.18	4.0×10^{-2}	0.54	2.9×10^{-11}	0.49	1.9×10^{-7}	1.12	(0.99-1.27)	6.8×10^{-2}
CXCL6	99	P80162	30.52	15625	CXCL6	50.36	3.8×10^{-18}	0.34	7.1×10^{-5}	0.25	4.7×10^{-3}	0.58	5.7×10^{-13}	0.41	2.4×10^{-5}	1.05	(0.93-1.2)	4.1×10^{-1}

CXCL9	100	Q07325	0.95	3906	CXCL9	31.43	1.3×10^{-17}	-0.01	9.3×10^{-1}	0.00	9.8×10^{-1}	-0.19	3.4×10^{-2}	-0.09	3.7×10^{-1}	0.99	(0.83-1.17)	8.7×10^{-1}
CXCL10	100	P02778	7.63	15625	CXCL9	8.36	5.6×10^{-15}	0.02	8.2×10^{-1}	-0.11	2.1×10^{-1}	-0.16	7.6×10^{-2}	0.00	9.6×10^{-1}	1.06	(0.77-1.45)	7.1×10^{-1}
CXCL11	99	O14625	30.52	15625	MCP-2	118.5	9.3×10^{-19}	0.30	4.4×10^{-4}	0.32	2.1×10^{-4}	0.49	3.3×10^{-9}	0.26	7.6×10^{-3}	0.95	(0.86-1.04)	2.7×10^{-1}
DNER	100	Q8NFT8	1.91	31250	LIF-R	0.71	2.0×10^{-7}	-0.18	3.8×10^{-2}	-0.05	5.7×10^{-1}	0.06	5.2×10^{-1}	0.03	7.3×10^{-1}	0.93	(0.58-1.5)	7.6×10^{-1}
4E-BP1	100	Q13541	-	-	STAMPB	94.83	7.2×10^{-19}	0.34	7.8×10^{-5}	0.10	2.4×10^{-1}	0.64	2.2×10^{-16}	0.47	7.1×10^{-7}	1.04	(0.91-1.18)	5.6×10^{-1}
EN-RAGE	97	P80511	122.07	500000	STAMPB	11.43	8.8×10^{-15}	0.40	3.2×10^{-6}	0.10	2.7×10^{-1}	0.55	9.0×10^{-12}	0.53	1.0×10^{-8}	1.13	(1-1.28)	4.9×10^{-2}
FGF-19	100	O95750	7.63	15625	BetaNGF	2.03	1.2×10^{-7}	0.18	4.3×10^{-2}	0.20	2.3×10^{-2}	0.66	2.2×10^{-16}	0.46	1.4×10^{-6}	1.19	(0.92-1.53)	1.9×10^{-1}
FGF-21	62	Q9NSA1	30.52	62500	NA	NA	NA	NA	NA	NA	NA	NA	NA	NA	NA	NA	NA	NA
FGF-23	20	Q9GZV9	122.07	62500	NA	NA	NA	NA	NA	NA	NA	NA	NA	NA	NA	NA	NA	NA
FGF-5	100	Q8NF90	1.91	31250	FGF5	0.53	1.2×10^{-7}	-0.23	7.6×10^{-3}	-0.20	2.0×10^{-2}	-0.33	1.3×10^{-4}	-0.20	4.9×10^{-2}	0.87	(0.62-1.23)	4.3×10^{-1}
Flt3L	100	P49771	0.01	977	Flt3L	1.36	3.4×10^{-4}	-0.11	2.1×10^{-1}	-0.10	2.8×10^{-1}	0.11	2.1×10^{-1}	0.10	3.2×10^{-1}	1.13	(0.83-1.53)	4.5×10^{-1}
GDNF	15	P39905	0.01	1953	NA	NA	NA	NA	NA	NA	NA	NA	NA	NA	NA	NA	NA	NA
HGF	100	P14210	7.63	125000	BetaNGF	4.40	6.7×10^{-18}	0.34	9.7×10^{-5}	0.24	6.9×10^{-3}	0.71	2.2×10^{-16}	0.65	2.7×10^{-13}	1.38	(1.07-1.79)	1.5×10^{-2}
IFN-γ	91	P01579	15.26	62500	MCP-2	33.69	2.3×10^{-18}	0.26	2.4×10^{-3}	0.21	1.9×10^{-2}	0.50	2.0×10^{-9}	0.23	2.1×10^{-2}	0.91	(0.81-1.01)	9.0×10^{-2}
IL-1α	44	P01583	0.95	31250	NA	NA	NA	NA	NA	NA	NA	NA	NA	NA	NA	NA	NA	NA
IL-1RA	92	P18510	780	100000	STAMPB	13.89	8.0×10^{-10}	0.40	3.3×10^{-5}	0.11	2.8×10^{-1}	0.69	1.3×10^{-15}	0.39	5.8×10^{-5}	0.96	(0.82-1.12)	5.8×10^{-1}
IL-2	3	P60568	30.52	1000000	NA	NA	NA	NA	NA	NA	NA	NA	NA	NA	NA	NA	NA	NA
IL-2RB	2	P14784	30.52	250000	NA	NA	NA	NA	NA	NA	NA	NA	NA	NA	NA	NA	NA	NA
IL-10	92	P22301	0.48	62500	CXCL5	27.23	1.6×10^{-18}	0.36	2.2×10^{-5}	0.24	5.8×10^{-3}	0.61	8.9×10^{-15}	0.32	9.7×10^{-4}	0.99	(0.85-1.16)	9.5×10^{-1}
IL-10RA	67	Q13651	7.63	250000	NA	NA	NA	NA	NA	NA	NA	NA	NA	NA	NA	NA	NA	NA
IL-10RB	100	Q08334	0.12	1953	STAMPB	3.47	4.4×10^{-17}	0.20	2.2×10^{-2}	0.05	5.9×10^{-1}	0.79	2.2×10^{-16}	0.58	2.8×10^{-10}	1.49	(1.21-1.84)	2.0×10^{-4}
IL-12B	98	P29460	0.12	3906	IL-12B	18.10	8.3×10^{-19}	0.17	6.0×10^{-2}	0.33	1.5×10^{-4}	0.57	1.5×10^{-12}	0.31	1.5×10^{-3}	0.86	(0.74-0.99)	3.5×10^{-2}
IL-13	54	P35225	7.63	62500	NA	NA	NA	NA	NA	NA	NA	NA	NA	NA	NA	NA	NA	NA
IL-15RA	86	Q13261	0.95	7812	STAMPB	1.53	2.0×10^{-16}	0.15	8.7×10^{-2}	0.12	1.6×10^{-1}	0.71	2.2×10^{-16}	0.46	1.2×10^{-6}	1.74	(1.15-2.64)	8.6×10^{-3}
IL-17A	67	Q16552	7.63	62500	NA	NA	NA	NA	NA	NA	NA	NA	NA	NA	NA	NA	NA	NA
IL-17C	2	Q9P0M4	30.52	125000	NA	NA	NA	NA	NA	NA	NA	NA	NA	NA	NA	NA	NA	NA
IL-18	100	Q14116	0.06	15625	STAMPB	17.48	8.7×10^{-18}	0.31	2.7×10^{-4}	0.12	1.7×10^{-1}	0.75	2.2×10^{-16}	0.53	1.1×10^{-8}	1.24	(1.05-1.47)	1.2×10^{-2}
IL-18R1	100	Q13478	0.06	7812	STAMPB	7.23	1.1×10^{-18}	0.34	7.4×10^{-5}	0.19	2.9×10^{-2}	0.74	2.2×10^{-16}	0.59	5.6×10^{-11}	1.3	(1.05-1.6)	1.5×10^{-2}

IL-20	1	Q9NYY1	15.26	62500	NA	NA	NA	NA	NA	NA	NA	NA	NA	NA	NA	NA	NA	NA
IL-20RA	2	Q9UHF4	1.91	125000	NA	NA	NA	NA	NA	NA	NA	NA	NA	NA	NA	NA	NA	NA
IL-22 RA1	0	Q8N6P7	0.24	3906	NA	NA	NA	NA	NA	NA	NA	NA	NA	NA	NA	NA	NA	NA
IL-24	32	Q13007	3.81	31250	NA	NA	NA	NA	NA	NA	NA	NA	NA	NA	NA	NA	NA	NA
IL-33	0	O95760	3.81	31250	NA	NA	NA	NA	NA	NA	NA	NA	NA	NA	NA	NA	NA	NA
IL-4	4	P05112	0.24	7812	NA	NA	NA	NA	NA	NA	NA	NA	NA	NA	NA	NA	NA	NA
IL-5	5	P05113	3.81	15625	NA	NA	NA	NA	NA	NA	NA	NA	NA	NA	NA	NA	NA	NA
IL-6	100	P05231	0.12	3906	OSM	185.4	5.4×10^{-18}	0.42	7.1×10^{-7}	0.15	8.0×10^{-2}	0.40	1.9×10^{-6}	0.30	2.6×10^{-3}	0.98	(0.89-1.07)	6.6×10^{-1}
IL-7	80	P13232	0.24	7812	IL-7	1.21	2.9×10^{-2}	0.13	1.3×10^{-1}	-0.33	1.3×10^{-4}	0.09	2.9×10^{-1}	0.28	4.9×10^{-3}	1.6	(1.16-2.21)	3.9×10^{-3}
IL-8	100	P10145	0.03	3906	IL-8	17.11	1.3×10^{-18}	0.30	6.3×10^{-4}	0.22	1.2×10^{-2}	0.33	1.5×10^{-4}	0.19	6.3×10^{-2}	0.98	(0.82-1.18)	8.6×10^{-1}
LAP TGF-β1	100	P01137	61.04	500000	STAMPB	3.84	6.5×10^{-18}	0.28	1.1×10^{-3}	0.13	1.4×10^{-1}	0.72	2.2×10^{-16}	0.61	8.9×10^{-12}	1.55	(1.23-1.96)	2.0×10^{-4}
LIF	95	P15018	7.63	15625	OSM	15.79	1.6×10^{-18}	0.49	2.5×10^{-9}	0.27	2.0×10^{-3}	0.51	7.1×10^{-10}	0.42	1.2×10^{-5}	1.13	(0.97-1.31)	1.2×10^{-1}
LIF-R	95	P42702	15.26	62500	LIF-R	0.62	1.2×10^{-8}	-0.09	3.2×10^{-1}	0.03	7.7×10^{-1}	0.06	4.7×10^{-1}	0.16	1.0×10^{-1}	1.17	(0.72-1.91)	5.2×10^{-1}
MCP-1	100	P13500	0.03	1953	MCP-2	4.01	8.9×10^{-16}	0.33	1.5×10^{-4}	0.18	4.5×10^{-2}	0.53	1.0×10^{-10}	0.27	7.1×10^{-3}	0.83	(0.65-1.05)	1.1×10^{-1}
MCP-2	100	P80075	0.06	3906	MCP-2	184.7	7.9×10^{-19}	0.35	5.5×10^{-5}	0.25	4.9×10^{-3}	0.54	2.2×10^{-11}	0.27	5.4×10^{-3}	0.93	(0.85-1.02)	1.1×10^{-1}
MCP-3	98	P80098	0.48	1953	MCP-2	129.8	7.2×10^{-19}	0.36	2.6×10^{-5}	0.25	4.2×10^{-3}	0.52	2.1×10^{-10}	0.34	4.4×10^{-4}	0.95	(0.86-1.04)	2.6×10^{-1}
MCP-4	81	Q99616	7.63	3906	MCP-4	2.05	3.0×10^{-15}	0.34	7.3×10^{-5}	0.19	3.2×10^{-2}	0.43	3.6×10^{-7}	0.39	4.8×10^{-5}	1.28	(1.03-1.59)	2.5×10^{-2}
MIP-1α	100	P10147	0.06	488	STAMPB	30.64	7.2×10^{-19}	0.44	1.4×10^{-7}	0.19	3.3×10^{-2}	0.69	2.2×10^{-16}	0.43	6.4×10^{-6}	1.01	(0.87-1.18)	8.5×10^{-1}
MMP-1	100	P03956	3.81	15625	OSM	19.58	1.5×10^{-17}	0.43	3.1×10^{-7}	0.24	6.5×10^{-3}	0.53	7.5×10^{-11}	0.35	3.6×10^{-4}	1.07	(0.94-1.21)	3.1×10^{-1}
MMP-10	100	P09238	0.95	15625	MMP-10	4.17	3.9×10^{-14}	0.42	4.9×10^{-7}	0.30	5.6×10^{-4}	0.57	1.2×10^{-12}	0.48	4.0×10^{-7}	1.31	(1.08-1.6)	6.1×10^{-3}
NRTN	3	Q99748	7.63	15625	NA	NA	NA	NA	NA	NA	NA	NA	NA	NA	NA	NA	NA	NA
NT-3	2	P20783	0.12	3906	NA	NA	NA	NA	NA	NA	NA	NA	NA	NA	NA	NA	NA	NA
OPG	100	O00300	0.48	31250	OPG	0.86	1.8×10^{-1}	0.18	4.2×10^{-2}	0.08	3.7×10^{-1}	0.32	1.8×10^{-4}	0.43	6.5×10^{-6}	1.49	(1.18-1.88)	6.9×10^{-4}
OSM	95	P13725	0.03	977	OSM	67.37	7.2×10^{-19}	0.48	7.8×10^{-9}	0.20	2.6×10^{-2}	0.44	1.3×10^{-7}	0.38	8.7×10^{-5}	1.02	(0.91-1.14)	7.2×10^{-1}
PD-L1	100	Q9NZQ7	3.81	500000	STAMPB	14.51	8.3×10^{-19}	0.29	7.2×10^{-4}	0.18	4.4×10^{-2}	0.70	2.2×10^{-16}	0.48	3.9×10^{-7}	1.05	(0.87-1.26)	6.0×10^{-1}
S100A8/A9	100	-	93760	12000000	STAMPB	6.36	2.5×10^{-10}	0.53	1.4×10^{-8}	0.21	3.6×10^{-2}	0.55	1.9×10^{-9}	0.51	6.9×10^{-8}	1.1	(0.94-1.28)	2.4×10^{-1}
SCF	100	P21583	3.81	15625	BetaNGF	1.70	1.4×10^{-6}	0.17	5.7×10^{-2}	0.15	9.2×10^{-2}	0.67	2.2×10^{-16}	0.47	8.7×10^{-7}	1.35	(1.08-1.7)	1.0×10^{-2}
SIRT2	98	Q8IXJ6	15.26	62500	STAMPB	11.60	8.8×10^{-16}	0.34	9.3×10^{-5}	0.08	3.8×10^{-1}	0.69	2.2×10^{-16}	0.56	9.2×10^{-10}	1.15	(1.02-1.3)	2.1×10^{-2}

SLAMF1	88	Q13291	30.52	1000000	STAMPB	3.51	8.5×10^{-18}	0.25	3.6×10^{-3}	0.18	3.7×10^{-2}	0.79	2.2×10^{-16}	0.50	8.7×10^{-8}	1.28	(1.06-1.54)	9.2×10^{-3}
ST1A1	79	P50225	244.14	125000	ST1A1	3.02	5.2×10^{-15}	0.36	3.0×10^{-5}	0.18	4.5×10^{-2}	0.46	4.8×10^{-8}	0.31	1.5×10^{-3}	1.07	(0.88-1.3)	5.1×10^{-1}
STAMPB	92	O95630	7.63	31250	STAMPB	7.45	1.6×10^{-18}	0.37	1.8×10^{-5}	0.14	1.2×10^{-1}	0.66	2.2×10^{-16}	0.53	1.1×10^{-8}	1.16	(0.98-1.36)	7.7×10^{-2}
TGF-α	100	P01135	0.48	3906	TGF-α	0.95	2.5×10^{-1}	0.21	1.7×10^{-2}	0.00	9.8×10^{-1}	0.23	7.4×10^{-3}	0.22	3.0×10^{-2}	1.51	(0.97-2.36)	6.8×10^{-2}
TNF	79	P01375	0.48	3906	STAMPB	3.98	1.8×10^{-15}	0.33	1.2×10^{-4}	0.20	2.5×10^{-2}	0.62	2.2×10^{-15}	0.38	8.6×10^{-5}	1.01	(0.87-1.17)	9.4×10^{-1}
TNFB	94	P01374	0.48	15625	MCP-2	50.53	1.5×10^{-18}	0.22	1.1×10^{-2}	0.27	2.2×10^{-3}	0.56	5.2×10^{-12}	0.29	3.3×10^{-3}	0.91	(0.81-1.02)	1.1×10^{-1}
TNFRSF9	100	Q07011	0.03	3906	STAMPB	37.70	7.2×10^{-19}	0.25	4.6×10^{-3}	0.11	2.3×10^{-1}	0.71	2.2×10^{-16}	0.43	7.7×10^{-6}	1.09	(0.92-1.3)	3.1×10^{-1}
TNFSF14	95	O43557	1.91	15625	MCP-2	11.81	7.2×10^{-19}	0.40	3.0×10^{-6}	0.37	1.7×10^{-5}	0.55	1.0×10^{-11}	0.33	8.7×10^{-4}	0.88	(0.74-1.04)	1.3×10^{-1}
TRAIL	100	P50591	0.95	31250	STAMPB	11.04	7.2×10^{-19}	0.38	8.1×10^{-6}	0.21	1.4×10^{-2}	0.69	2.2×10^{-16}	0.43	6.9×10^{-6}	0.99	(0.82-1.2)	9.4×10^{-1}
TRANCE	21	O14788	3.81	31250	NA	NA	NA	NA	NA	NA	NA	NA	NA	NA	NA	NA	NA	NA
TSLP	0	Q969D9	3.81	15625	NA	NA	NA	NA	NA	NA	NA	NA	NA	NA	NA	NA	NA	NA
TWEAK	100	O43508	1.91	125000	TWEAK	0.88	1.6×10^{-1}	-0.04	6.4×10^{-1}	-0.12	1.7×10^{-1}	0.33	1.4×10^{-4}	0.41	2.0×10^{-5}	1.93	(1.37-2.72)	1.6×10^{-4}
uPA	100	P00749	0.12	7812	STAMPB	8.77	2.4×10^{-18}	0.41	1.7×10^{-6}	0.19	3.5×10^{-2}	0.71	2.2×10^{-16}	0.53	8.8×10^{-9}	1.15	(0.96-1.38)	1.4×10^{-1}
VEGF-A	100	P15692	0.06	7812	VEGFA	2.49	2.0×10^{-11}	0.28	1.1×10^{-3}	0.14	1.2×10^{-1}	0.57	1.0×10^{-12}	0.51	4.5×10^{-8}	1.57	(1.24-1.99)	1.6×10^{-4}

Abbreviations: FC = fold change; Corr. = Spearman correlation; CSF = cerebrospinal fluid; MN = mononuclear cells; PMN = polymorphonuclear cells; NA = not applicable.

Supplementary Table 4. Cerebrospinal fluid MMP-10 quantitative trait loci in tuberculous meningitis

SNP	Chr	Position	Ref allele	Minor allele	Beta	p-value	Nearest gene
rs16940337	18	13302390	G	A	1.25	8.04×10^{-9}	LDLRAD4 (intronic)
rs950793	8	26671993	G	T	-1.04	4.80×10^{-8}	ADRA1A (intronic)
rs10741857	11	20899163	A	G	-1.09	8.01×10^{-7}	NELL1 (intronic)
rs10794798	10	1932908	C	G	0.83	5.81×10^{-6}	Intergenic
rs12491058	3	59542556	G	A	1.01	4.99×10^{-6}	FHIT
rs9871763	3	62477699	C	A	1.03	7.71×10^{-6}	CADPS (intronic)

Chr = chromosome; Ref allele = Reference allele.

Supplementary Table 5. MMP-10 quantitative trait loci in protein cluster representatives associated with mortality

Protein	SNP	Beta	p-value
TWEAK	rs16940337	-0.59	1.69×10^{-7}
	rs950793	0.076	0.457643
	rs10741857	0.543	1.99×10^{-6}
	rs10794798	-0.162	0.093117
	rs12491058	-0.595	1.55×10^{-7}
	rs9871763	0.121	0.321783
VEGF	rs16940337	-0.051	0.714959
	rs950793	-0.455	0.000178
	rs10741857	0.094	0.504483
	rs10794798	0.282	0.01453
	rs12491058	-0.258	0.065767
	rs9871763	0.336	0.020539
OPG	rs16940337	0.298	0.044314
	rs950793	-0.459	0.000393
	rs10741857	-0.215	0.150261
	rs10794798	0.056	0.649964
	rs12491058	0.202	0.176687
	rs9871763	0.585	0.00013
IL-7	rs16940337	0.616	6.43×10^{-9}
	rs950793	-0.298	0.001974
	rs10741857	-0.595	3.05×10^{-8}
	rs10794798	0.153	0.095077
	rs12491058	0.537	6.57×10^{-7}
	rs9871763	0.172	0.135472
MMP-10	rs16940337	1.247	8.04×10^{-9}
	rs950793	-1.045	4.80×10^{-8}
	rs10741857	-1.086	8.01×10^{-7}
	rs10794798	0.825	5.81×10^{-6}
	rs12491058	1.007	4.99×10^{-6}
	rs9871763	1.027	7.71×10^{-6}
